# Early Rectal Cancer and Local Excision: A Narrative Review

**DOI:** 10.3390/jcm13082292

**Published:** 2024-04-16

**Authors:** Cecilia Binda, Matteo Secco, Luigi Tuccillo, Chiara Coluccio, Elisa Liverani, Carlo Felix Maria Jung, Carlo Fabbri, Giulia Gibiino

**Affiliations:** Gastroenterology and Digestive Endoscopy Unit, Forlì-Cesena Hospitals, AUSL Romagna, 47121 Forlì, Italy; cecilia.binda@auslromagna.it (C.B.); matteo.secco@auslromagna.it (M.S.); luigi.tuccillo@studio.unibo.it (L.T.); chiara.coluccio@auslromagna.it (C.C.); elisa.liverani@auslromagna.it (E.L.); carlofelixmaria.jung@auslromagna.it (C.F.M.J.); giulia.gibiino@auslromagna.it (G.G.)

**Keywords:** rectal cancer, early rectal cancer, local excision, transanal endoscopic surgery, local treatment

## Abstract

A rise in the incidence of early rectal cancer consequent to bowel-screening programs around the world and an increase in the incidence in young adults has led to a growing interest in organ-sparing treatment options. The rectum, being the most distal portion of the large intestine, is a fertile ground for local excision techniques performed with endoscopic or surgical techniques. Moreover, the advancement in endoscopic optical evaluation and the better definition of imaging techniques allow for a more precise local staging of early rectal cancer. Although the local treatment of early rectal cancer seems promising, in clinical practice, a significant number of patients who could benefit from local excision techniques undergo total mesorectal excision (TME) as the first approach. All relevant prospective clinical trials were identified through a computer-assisted search of the PubMed, EMBASE, and Medline databases until January 2024. This review is dedicated to endoscopic and surgical local excision in the treatment of early rectal cancer and highlights its possible role in current and future clinical practice, taking into account surgical completion techniques and chemoradiotherapy.

## 1. Introduction

The implementation of bowel-screening programs around the world and the increasing awareness of the population have promoted a shift towards the detection of rectal cancer in earlier stages [1,2].

Nonetheless, colorectal cancer is still reported as the second most common cause of cancer death in the United States, with around 46,000 new diagnoses of rectal cancer in the USA in 2023 [3]. Moreover, while the global incidence of CRC has slowly declined over the last 20 years, an increase in the incidence in young adults (age < 50 years) has been observed [4]. In 2023, in the USA, 7000 new people below 50 years old received a diagnosis of rectal cancer, and these data are even more important if we consider that this subset of patients has been shown to have a worse prognosis, at every rectal cancer stage, compared to the older population [3,5].

Early rectal cancer can be defined as a lesion localized to the rectal wall in which the likelihood of mesorectal disease, nodal positivity, or deposits is low, and, correspondingly, the risk of recurrence after local excision is at an acceptable level [6].

Surgery as total mesorectal excision (TME) is the cornerstone of treatment in patients with rectal cancer; the algorithm of treatment in these patients varies in accordance to the disease stage, patient clinical status, and patient’s preference, but most cases of early-stage rectal cancer (defined as T1–2 and node negativity) are managed by upfront surgery, while, in the case of local advanced rectal cancer (defined as T3–4 and/or nodal positivity), neoadjuvant chemoradiotherapy (CRT) before surgery is recommended [7].

Radical TME surgery allows the dissection of the tumor and mesorectum with all the associated lymph nodes through the avascular embryologic plane but is burdened by adverse events and side effects like fecal incontinence, urinary and sexual dysfunction, and stoma rates [8,9,10]. 

On the other hand, local excision techniques has been associated to inferior oncologic outcomes compared to radical surgery, but, with the appropriate patient selection, they may provide an oncological radical excision with less morbidity and functional impairment, and, therefore, better outcomes in terms of quality of life [11,12,13].

The increasing amount of rectal cancer detected in the early stages and the availability of techniques able to obtain the complete excision of the tumor sparing the rectum are changing this paradigm, making local excision techniques the first approach for an increasing number of patients [14,15].

In this narrative review, we will focus on early rectal cancer diagnosis, local staging, management, and future perspectives.

## 2. Materials and Methods

We selected articles discussing the topic of early rectal cancer and its treatment, paying specific attention to surgical or endoscopic local excision for rectal cancer in early stages. We developed a non-systematic review article using the following electronic sources: PubMed, EMBASE, Google Scholar, Ovid, MEDLINE, Scopus, the Cochrane controlled trials register, and Web of Science. We used the following search terms alone and in combination: “Rectal Neoplasms”, “Minimally Invasive Surgical Procedures”, “Transanal Endoscopic Surgery”, “endoscopic resection”, “T1”, “pT1”, “T2”, “pT2”, “early rectal cancer”, “early colorectal cancer”, “chemotherapy”, “radiotherapy”, “low risk rectal cancer”, “high risk rectal cancer”, “local treatment”, “local excision”, “FTR”, “endoscopic submucosal dissection”, “full thickness resection”, “TAMIS”, and “TEM”. We examined all the articles reporting data related to humans (inclusion criterion) while excluding works with no full text available, works that were not in the English language, book chapters and abstracts, and articles published before 1990. Finally, we evaluated supplementary references among the articles evaluated in the first search round.

## 3. Staging

The most frequently used staging method for rectal cancer is the TNM classification [16]. Considering early-stage rectal cancer (defined as T1–2 and node negativity) Tis refers to intramucosal adenocarcinoma, T1 applies to rectal tumors with submucosal infiltration while T2 shows extension to the muscularis propria. 

Pre-operative staging of early rectal lesions is crucial in establishing the right intervention strategy. Staging outside of the pelvis is usually obtained with computed tomography scan of thorax and abdomen with addition of PET in selected patients [17]. Endoscopic optic evaluation, magnetic resonance imaging (MRI), and endoscopic rectal ultrasound (ERUS) are the most accurate methods to define locoregional clinical staging.

### 3.1. Endoscopic Optic Evaluation

Endoscopic optic evaluation, through dye chromoendoscopy and image-enhanced endoscopy such as narrow-band imaging (NBI), can be used to identify deep submucosal invasion as soon as the lesion is detected [18,19]. Morphologic characteristics such as lesion size, location, spontaneous bleeding, ulceration, the Paris classification system, and, eventually, the non-lifting sign provide crucial information to predict the chance of curative endoscopic resection of the lesion and the risk of covert submucosal invasive cancer [19,20]. Sensitivity of morphologic features alone as an indicator of T1 tumor has been reported to be quite low in several studies [21,22]. Advanced imaging techniques such as chromoendoscopy and narrow-band imaging (NBI) enhanced the endoscopist’s ability to diagnose T1 tumors and to identify submucosal invasion [23]. Classifications based on the use of advanced imaging techniques such as NICE or JNET provide tools for a correct stratification of the risk of shallow submucosal invasive cancer and deep submucosal invasive cancer [24]. However, diagnostic accuracy of these classifications is operator-dependent, and it is reported to range from 59.5% to 84.2% [25]. Chromoendoscopy uses stains or dyes during endoscopy to improve the visualization and characterization of the gastrointestinal mucosa and to assess lesion architecture and pit pattern; this technique is widely available but it is time-consuming since the dye must be sprayed all over the lesion before the evaluation [26]. Narrow-band imaging is a filter that can be used during endoscopic evaluation simply by pressing a button on the endoscope. The use of this filter allows better evaluation of the lesion surface, assessing mucosal microcapillaries and their modifications. The advantage of this technique is the prompt availability without the need for spraying die over the lesion. Moreover, many studies have reported that this technique is equivalent to chromoendoscopy with dye in terms of accuracy for diagnosis of submucosal invasion [27]. Chromoendoscopy can differentiate CRC lesions with deep submucosal invasion from lesions with or without submucosal invasion (polyp, adenoma, dysplasia, intramucosal cancer, or submucosal invasive cancer) with high accuracy and it can guide assessment of invasion depth of submucosa in T1 early CRC [25]. However, a small risk of submucosal invasive cancer (SMIC), even with accurate endoscopic lesion evaluation, is always present. In the near future, we can expect that new advanced imaging techniques and the introduction of artificial intelligence will further enhance optical evaluation diagnostic accuracy. 

### 3.2. MRI

The gold standard for local staging of rectal cancer is magnetic resonance imaging (MRI). Rectal MRI helps to predict the risk of recurrence and distant metastases by providing cT substage, relation of the tumor to the mesorectal fascia, extramural vascular invasion, and pathologic lymph nodes [17]. However, MRI is unable to differentiate between T1 (submucosal infiltration) and T2 (extension to the muscularis propria) stages in early rectal cancer because of the submucosal layer distortion caused by the rectal lesion [28]. These data are even more important if we consider that, in a population study by Detering et al. among patients with pT1 tumors, 54.7 per cent (792 of 1448) were overstaged by MRI, precluding in them the possibility of local excision [28]. In addition, MRI showed limits also in assessing nodal disease, with sensitivity as low as 28.6% for nodal disease in patients with pT1 rectal cancer [29]. The risk of occult lymph node metastasis ranges from 5–10% in stage T1 to 20–35% in stage T2 tumours [30]. High-resolution MRI is reported to be able to overcome the limit of differentiation between T1 and T2 but its availability and the need of radiologist specialized in early rectal cancer are limiting its use in clinical practice [31]. MRI for local staging has also been considered in combination with 18F-fluorodeoxyglycose positron emission tomography (FDG-PET). FDG-PET/MRI can help to better delineate the extent of tumor and is particularly useful in evaluating the presence of tumor extension beyond the muscularis propria [32,33]. Moreover, the combination of PET and MRI has shown potential for local nodal staging for rectal cancer since hypermetabolism on PET appears to have a higher specificity than MRI, particularly for small nodes and can, therefore, help to better characterize small pelvic nodes [34]. Characterization of small pelvic nodes and evaluation of extension beyond the muscularis propria are both important features to evaluate which patients should be approached with local excision techniques, making PET/MRI an interesting combination technique for this subset of patients.

### 3.3. ERUS

Endoscopic rectal ultrasound (ERUS) is a technique that allows a better clinical staging of rectal cancer and which can be used in combination with MRI to overcome MRI limits [35]. ERUS must be performed by expert clinicians since it is a highly operator-dependent technique. In a recent meta-analysis by Luglio G. et al., ERUS outperformed MRI in all T stages except T4, with remarkable difference in T1 tumors; T4 rectal cancer can benefit from MRI, which, at this stage, is connoted by higher sensitivity and slightly lower specificity [36]. ERUS also outperforms MRI in advanced T3 tumors as reported by Chan et al. due to a higher sensitivity for small lesion and a better visualization of thin layers such as the serosa [37]. For the evaluation of nodal involvement, different meta-analyses suggest a similar or slightly more accurate nodal staging with ERUS with respect to MRI [36,37]. Contrast-enhanced endoscopic ultrasound (CE-EUS) is not routinely performed in evaluation and local staging of rectal cancer; however, in a small case series by Gibiino et al. including 12 rectal lesions, CE-EUS staging provided useful information regarding either the integrity of the muscular layer and the presence of vascularization, which are both factors known to be predictive of non-curative endoscopic resection; CE-EUS staging corresponded to the final pathological stages in 9/12 (75%) lesions, improving the distinction between T1 and T2 lesions [38]. ERUS and MRI are complementary imaging techniques with different limits and advantages; furthermore, their combination in the context of early rectal cancer leads to a lower percentage of overstaging, although this percentage remained as high as 31% [28].

## 4. Surgical and Endoscopic Techniques

### 4.1. Total Mesorectal Excision (TME)

Total mesorectal excision (TME) stands as the cornerstone of curative-intent therapy, involving the removal of the affected rectum alongside the mesorectum, encompassing vascular and lymphatic structures, adipose tissue, and mesorectal fascia, all in one ‘tumor package’ [8,39]. The delineation of the tissue package’s boundaries relies on dissection along embryological planes [8]. The appropriate plane for dissection lies in an avascular and areolar tissue plane between the mesorectal fascia and the parietal pelvic fascia, commonly known as the ‘holy plane’ [13]. For mid- to upper rectal tumors, TME can be achieved through low anterior resection (LAR), followed by colorectal or coloanal anastomosis [39]. A recommended distal margin of 5 cm applies to rectal tumors in the upper third of the rectum according to the American Society of Colon and Rectal Surgeons (ASCRS) 2020 Clinical Practice Guidelines [40]. For mid- to lower-third rectal tumors, a 2 cm distal margin suffices for low colorectal anastomosis, while at least a 1 cm distal margin suffices for tumors at or below the mesorectal margin. For the lowest rectal tumors, especially those involving the anal sphincter or levator muscles, abdominoperineal resection (APR) is necessary to achieve TME [39]. Abdominoperineal resection (APR) entails the removal of the rectum, anus, mesorectum, and perianal soft tissues with the establishment of a permanent colostomy. Although TME is the gold standard in rectal cancer surgery, it carries significant potential morbidity: up to 80 to 90% of patients undergoing sphincter-sparing surgery for rectal cancer experience some degree of LAR syndrome, which encompasses symptoms ranging from incontinence with frequency and urgency to constipation and incomplete emptying [41]. However, the introduction of nerve-sparing mesorectal excision has reduced long-term urinary dysfunction rates from 26% to as low as 4% [42]. Simultaneously, the preservation of autonomic nerves aims to mitigate urinary and sexual dysfunction. Although sexual dysfunction is a known complication of proctectomy, the poor reporting in the literature obscures the true incidence of the complication [43]. Nevertheless, with the increase in rectal cancer diagnoses among individuals under 50 [44], post-proctectomy sexual dysfunction is likely to emerge as a more significant concern. Given the complications of TME, stakeholders are actively pursuing opportunities to minimize the morbidity of rectal cancer care without compromising the oncologic outcomes, leading to the introduction of various advancements in surgical care [13].

### 4.2. Local Excision Techniques 

The local excision (LE) of rectal cancer includes many different approaches (surgical or endoscopic) united by the goal of the complete removal of the tumor itself, sparing the rectum; Some approaches, like endoscopic submucosal dissection (ESD) or endoscopic intermuscular dissection (EID), are not virtually influenced by lesion extension or position, but they do not offer a full-thickness resection of the tumor to the perirectal fat, while others can grant a full-thickness resection with, sometimes, the possibility of retrieving some perirectal nodes but can suffer from limits linked to lesion position or extension. During the last 20 years, several approaches to local excision have been used and are still being developed for both surgical and endoscopic techniques [11,45].

#### 4.2.1. Trans-Sacral (Kraske) or Trans-Sphincteric (York-Mason) Approaches

Local excision by posterior approaches has been used in the past and is, nowadays, largely abandoned. The trans-sacral approach (Kraske) carries an important complication rate and, thus, was used in managing patients with mid-rectal tumors not amenable to other treatment options [46]. These techniques had the advantage where perirectal nodes could be removed for histopathological examination without the need for a total mesorectal excision; however, they are associated with an unacceptable rate of fistula formation, wound breakdown, and incontinence [45].

#### 4.2.2. Transanal Excision (TAE)

Transanal excision (TAE) using a conventional retractor is a technique for treating early rectal cancer. The patient is positioned, orienting the lesion toward the floor, usually in a lithotomy or a jackknife position; the rectum is irrigated and a retractor is positioned to gently dilate the anus until a good visualization is obtained. Next, the lesion is marked by scoring the mucosa circumferentially with electrocautery, which is used to incise the full thickness of the rectum along the scored outline around the lesion. This technique is significantly limited by exposure and visibility, resulting in the difficulty in achieving high-quality oncological resections. Furthermore, lesions located in the proximal two-thirds of the rectum are not reachable by TAE [47]. A recent meta-analysis comparing TAE with transanal endoscopic microsurgery (TEM) showed that TEM has a higher rate of negative microscopic margins, a reduced rate of specimen fragmentation, and a reduced rate of lesion recurrence with no difference in postoperative complications in comparison with TAE, suggesting its oncological superiority [48]. 

#### 4.2.3. Transanal Endoscopic Microsurgery (TEM)

Transanal endoscopic microsurgery (TEM) is a minimally invasive procedure capable of performing full-thickness excision using a rigid, beveled proctoscope that is 4 cm in diameter and 12 to 20 cm in length, a laparoscopic camera, and modified laparoscopic instruments [11,47]. 

The proctoscope has a flat or beveled end with a diameter of 40 mm and a length of 12 or 20 cm. Depending on the location of the lesion, the patient may need to be in a prone position for anterior lesions, supine or lithotomy for posterior lesions, and right or left-sided tilt for lateral lesions. The pneumorectum is maintained by an insufflation system, while a roller pump drives the suction irrigation [47]. 

A wide resection to the perirectal fat must be achieved to try to achieve a curative treatment for T1 rectal cancers. In selected patient groups, as patients who are unfit for surgery, this procedure has been applied even for more advanced rectal cancers.

Fecal incontinence remains a possible complication with rectal bleeding, proctalgia, rectal stenosis, and pelvic inflammation or abscess [47]. One of the limits of TEM is linked to the rigid platform used to perform the procedure, resulting in harder patient positioning and setup compared to procedures using flexible platforms; TEM is also limited by longer learning curves. In a recent case-matched analysis by Stipa et al., transanal minimally invasive surgery (TAMIS) appeared to be technically easier and able to overcome the TEM disadvantages in terms of cost and operative time, complex patients positioning, and reproducibility [49].

#### 4.2.4. Transanal Minimally Invasive Surgery (TAMIS)

Transanal minimally invasive surgery (TAMIS) is one of the most recent surgical procedures for rectal cancer. After platform insertion, a pneumorectum at 10–12 mmHg is set and a hook-type monopolar electrocautery or the harmonic scalpel is used for dissection and coagulation [50].

Less positioning is required in TAMIS as the port allows for 360 degrees of movement and visualization. The patient is placed in the lithotomy position regardless of the orientation and location of the lesion. Perforation, urinary tract infection, subcutaneous emphysema, and haemorrhoid thrombosis are the most frequent complications [47,50]. One of the limits of TEM and TAMIS was linked to the fact that it was unclear if, in the case of early salvage total mesorectal excision (TME) for oncological purposes, the results of TAMIS or TEM + TME were equivalent to primary treatment with TME. Several studies showed that no difference is found in outcomes between patients with rectal cancer undergoing salvage TME after TEM or TAMIS vs. those undergoing primary TME, an important prerequisite to extend indications for local excision in rectal cancer [51,52].

#### 4.2.5. Endoscopic Submucosal Dissection (ESD)

Endoscopic submucosal dissection (ESD) is an endoscopic technique used to remove lesions within the rectum and other parts of the gastrointestinal tract. It was developed in the mid-1990s in Japan to resect early-stage GI tumours [53].

This endoscopic resection technique specifically dissects the tissue over muscularis propria using an electrosurgical knife. Basically, a colloid solution is injected and the mucosa is incised to provide access to the submucosa; after a circumferential mucosal incision, the submucosa is dissected below the mucosa specimen [54]. This technique allows the en bloc resection of lesions of virtually any size, with a low rate of adverse events and low recurrence rates, but it is a demanding technique, with a long learning curve and requiring dedicated devices. One of the limits of this technique is linked to its dissection plane (the submucosa) which allows the complete removal of superficial lesions (confined to the mucosa or in the first part of submucosa) but not of lesions with a deep submucosal invasion/invasion of the muscularis propria. Adverse events of ESD are intraprocedural bleeding with an average rate of around 10% in large case series and delayed bleeding [55]. Perforation rates during ESD for colorectal lesions are reported to be around 4–5% in centers with great experience [54]; however, most perforations can be managed endoscopically using through-the-scope clips, over-the-scope clips (OTSCs), or endoscopic suturing devices [56]. 

#### 4.2.6. Endoscopic Intermuscular Dissection (EID)

Endoscopic intermuscular dissection (EID) is a novel resection technique developed for rectal lesions that involves dissection in the intermuscular plane, the plane between the longitudinal (external) and circular (internal) muscle layer [57]. Dissection through the intermuscular space would enable the attainment of R0 deep resection margins for T1 rectal cancer with deep submucosal invasion, whilst securing the integrity of the rectal wall [57].

The perimeter of the lesion is marked using soft tip coagulation, and, then, submucosal lifting is performed. 

A submucosal incision is created at the oral side and at the anal side of the lesion; through the latter incision, a tunnel is created at the anal side using submucosal dissection, reaching the inner circular muscle, and then exposing the outer longitudinal muscle layer; the optimal countertraction to facilitate safe intermuscular dissection is obtained using gravity and traction devices. The oral incision is reached, making a tunnel under the lesion; then, the lateral margins are incised to complete the dissection. 

The most common postoperative problems are moderate perianal pain, rectal stenosis, delayed bleeding, and inflammatory response (fever, pain, elevated C-reactive protein, and perirectal air without a fluid collection). The learning curve for EID is demanding, so it is essential that the procedure is carried out by endoscopists experienced in ESD because, despite the intermuscular space being tangential to the rectal wall and the endoscope being stable, it is crucial to recognize the intermuscular space and to be able to define accurately the dissection planes beyond the submucosal space [58].

#### 4.2.7. Endoscopic Full-Thickness Resection (EFTR)

Endoscopic full-thickness resection (EFTR) is a technique for the resection of colorectal lesions; it represents an alternative for lesions that would have required a surgical approach because of non-lifting epithelial lesions due to severe fibrosis and scarring, subepithelial lesions (SELs) arising from the muscularis propria (MP), and lesions in locations difficult to access or at a high risk of adverse events (e.g., within a diverticulum) [59]. EFTR implies a resection through all layers of the GI wall with defect closure in the setting of a full-thickness resection. There are two main approaches to this: the standard EFTR which includes a full-thickness resection followed by defect closure, or prior clip-assisted EFTR that secures the gastrointestinal wall patency before resection [60]. Over-the-scope (OTS) clip-assisted EFTR is a “close then cut” technique that can provide a full-thickness resection of epithelial and subepithelial lesions throughout the GI tract, a safer alternative that involves securing the defect before resection [59]. Nonexposed colorectal eFTR is now considered an established endoscopic resection technique for complex colorectal lesions and has the advantage of resecting all layers of the bowel wall and an easier learning curve. Limitations of the FTRD system concerning a full-thickness resection are scarring, fibrosis, and the thickness of the intestinal wall, especially in the lower rectum. Another limitation of the FTRD system is linked to the lesion size because the whole lesion must fit the resection device to allow a complete resection. As reported by Zwager et al., in a case series of 1892 patients who underwent eFTR, the procedure is safe with a low overall AE rate of 11.3% and no AE-related mortality, while the severe AE rate requiring surgery was 2.2% [61]. 

## 5. Risk-Adapted Early Rectal Cancer Management 

**Figure 1:**
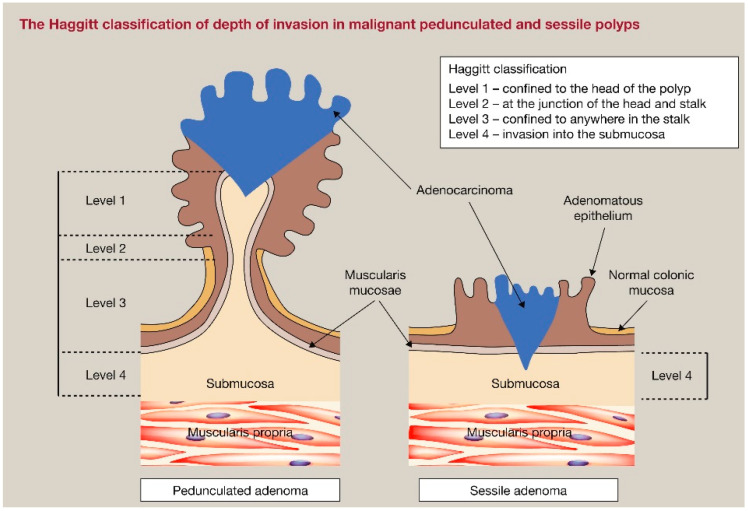
Haggitt classification for pedunculated and sessile lesions [67].

**Figure 2:**
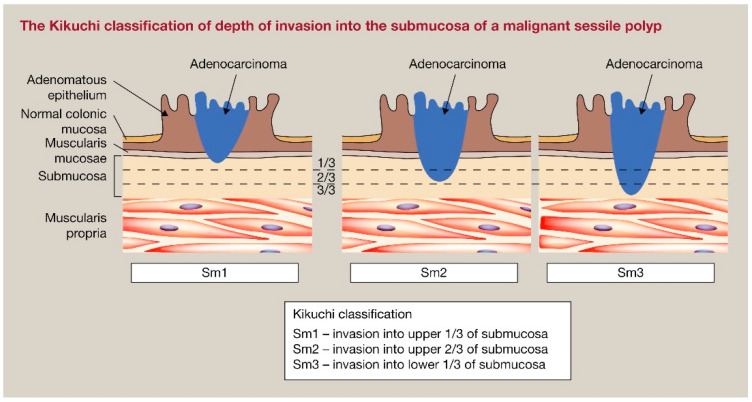
Kikuchi classification for sessile lesions [67].

Early-stage rectal cancer is defined as rectal cancer with the invasion of the submucosa or muscularis propria (cT1-2) and no nodal positivity, or extramural venous invasion (EMVI). Based on the combination of some microscopic and macroscopic features, we can distinguish between low-risk and high-risk early rectal cancers, and, thus, if a lesion might be safely removed, preserving the rectum [62]. One of these features is the degree of the submucosal invasion which can be evaluated with different classifications, taking into account the lesion morphology (pedunculated vs. non pedunculated). Haggitt et al. [63] proposed four different levels to stratify pedunculated lesions, ranging from 1 (invasion of submucosa limited to the head of the polyp) to 4 (invasion of submucosa beyond the stalk); this classification is still in use after almost 40 years for pedunculated lesions (Figure 1). The Kikuchi classification [64] aims to describe the depth of the submucosal invasion in non-pedunculated lesions, by dividing the submucosa in three different thirds: sm1 (first third of submucosa), sm2 (second third of submucosa), and sm3 (last third of submucosa) T1 cancers (Figure 2). Another criterion to evaluate the submucosal invasion is the one adopted by the Japanese Society for Cancer of the Colon and Rectum (JSCCR), which considers a submucosal invasion ≥ 1000 µm as a reliable feature of deep submucosal invasion. In the case of submucosal invasion ≥ 1000 µm, the lymph node metastasis rate is as high as 12.5% [65]. It is widely accepted to consider poorly differentiated adenocarcinoma, signet-ring cell carcinoma, mucinous carcinoma, lymphatic or vascular invasion, positive vertical margin, tumor budding (BD2/3 at the site of deepest invasion), and deep submucosal invasion (i.e., sm 2–3, Haggitt 4, or ≥1000 µm) as predictors of high-risk cancer [65,66]. 

### 5.1. Low-Risk Early Rectal Cancer

Rectal cancers cT1N0 without adverse features, such as poorly differentiated adenocarcinoma, signet-ring cell carcinoma, mucinous carcinoma, lymphatic or vascular invasion, positive vertical margin, tumor budding (BD2/3 at the site of the deepest invasion), and deep submucosal invasion (i.e., sm 2–3, Haggitt 4, or ≥1000 µm), are considered to be at a low risk of recurrence, and, thus, the treatment can be considered curative [65,68]. Treatment by local excision alone in this subset of patients allows the sparing of the rectum and all associated complications in the absence of significant differences in mortality and overall survival [17]; on the other hand, the most important limitation is the absence of the pathologic staging of nodal involvement [69]. Among all the possible local treatments, transanal endoscopic microsurgery seems to provide similar oncological results in pT1sm1 (clinical cN0) rectal cancers compared with results achieved by TME, without compromising anorectal function [17]. Another aspect that should be stressed is that not all the previously mentioned factors (i.e., histology, lymphatic or vascular invasion, positive vertical margin, tumor budding, and deep submucosal invasion) have the same role in predicting the risk of recurrence in T1 rectal cancer. According to recent reports, the frequency of lymph node metastasis (LNM) is about 1–2%, even with deep invasive cancer ≥ 1000 µm, as long as the other risk factors are negative [70,71]. In a case series from Yasue et al., the rate of LNM with only submucosal deep invasion as a risk factor was 1.6% (4/258), which was extremely low compared to the overall rate of LNM for T1 colorectal cancer in the previous reports (approximately 10%) [72]. Moreover, submucosal invasion depth (SID) measuring has shown several problems because SID is associated with lesion morphology, and it can sometimes become shorter in the progression of the lesions [73]. 

### 5.2. High-Risk Early Rectal Cancer

High-risk early rectal cancers are not suitable for local excision alone because of the high rate of local recurrence and mesorectal lymph node involvement [74].

Patients with T1N0M0 and the aforementioned risk factors at histopathological examination (poor differentiation, lymphatic or vascular invasion, positive vertical margin, tumor budding, and deep submucosal invasion) and patients with T2N0M0 must be included in the high-risk group. The standard of care for these patients is total mesorectal excision (TME), implying that all of the mesorectal fat, including all lymph nodes, should be meticulously excised. A partial mesorectal excision with a distal margin of at least 5 cm of the mesorectum can be considered in the high rectal cancer group. Laparoscopic, open, or robotic surgery is chosen based on the location of the lesion, the patient’s anatomical features, and the surgeon’s experience [17]. In patients with a high anesthesiologic risk, or who do not want to consider abdominoperineal resection, local excision treatment may still be proposed, preceded or adjuvanted by chemo- and radiotherapy treatment. This approach potentially decreases the risk of local and distant recurrence by sterilizing the mesorectal lymph nodes and the excision bed, with the expected lower morbidity and similar long-term survival [75]. In any case, watch-and-wait serious surveillance or chemotherapy treatment should follow radical surgery in high-risk early rectal cancers. For T2N0 < 4 cm in elderly/frail patients or patients not agreeing to undergo TME, local excision after preoperative radiotherapy/chemoradiotherapy has been considered; however, this strategy is not routinely recommended outside of clinical trials [17,76]. 

### 5.3. Worsening Restaging on Pathology after Local Excision 

If the pathology review after local excision reveals poorly differentiated adenocarcinoma, signet-ring cell carcinoma, mucinous carcinoma, lymphatic or vascular invasion, positive vertical margin, tumor budding (BD2/3 at the site of deepest invasion), and deep submucosal invasion (i.e., sm 2–3, Haggitt 4, or ≥1000 µm), or if the tumor is restaged to pT2, additional treatment is required [65,68]. Chemoradiotherapy protocols are possible in rectal-sparing strategies or in unfit patients. For patients treated with transanal local excision and then chemo-RT, the options for the next phase of treatment depend on whether there is evidence of residual disease. If there is no evidence of disease, observation or chemotherapy without resection may be considered. If there is evidence of disease, transabdominal resection should be performed, with or without adjuvant chemotherapy, because the local recurrence rate appeared to be higher in patients with locally excised pT1/pT2 category rectal cancer treated by adjuvant (chemo)radiotherapy than in patients who underwent completion TME [15].

## 6. Chemotherapy and Radiotherapy in Early Rectal Cancer

Early rectal cancer poses a complex challenge in terms of preserving organ function and ensuring favorable outcomes. 

Chemotherapy, radiotherapy, and their integration have raised the issue of offering patients with small residual cancers restricted to the bowel wall an alternative treatment strategy to total mesorectal excision even if not strictly indicated at first-time evaluation [77]. Neoadjuvant therapy is increasingly favored due to its potential to enhance tumor downstaging, improve surgical outcomes, and increase the likelihood of sphincter preservation. Adjuvant therapy may be considered in specific cases where neoadjuvant treatment is not feasible.

### 6.1. Neoadjuvant Therapy

The primary goal is to achieve tumor regression and facilitate surgical intervention with the intent of sphincter preservation. This approach allows for a more conservative treatment strategy, minimizing the impact on patients’ quality of life. Short-course preoperative radiotherapy (SCPRT) and chemoradiotherapy (CRT) are the standards of care for preoperative treatments; recent studies suggest that the results of both are similar. Despite this, in clinical practice, the chemoradiotherapy approach is preferred for high-risk lesions [76]. The most-used schedule for SCPRT is a 25 Gy total dose at 5 Gy/fraction during 1 week, followed by immediate surgery (<10 days from the first radiation fraction); SCPRT with delayed surgery is also a useful alternative to conventional short-course RT, with immediate surgery offering similar oncological outcomes and lower postoperative complications: CRT is based on a recommended dose of 45–50 Gy in 25–28 fractions; a boost with a further 5.4 Gy in 3 fractions can be considered for preoperative RT if the CRM is threatened, and for postoperative RT routinely with 5.4–9.0 Gy in 3–5 fractions according to CRM [17]. Other strategies could include the use of neoadjuvant capecitabine (725–825 mg/m^2^ on days 1–14 and 22–35) and oxaliplatin (50 mg/m^2^ in weeks 1, 2, 4, and 5) during radiotherapy, given to a total dose of 50–54 Gy, then followed by LE [14]. These protocols, designed to mostly precede surgical treatment with total mesorectal excision, are under investigation for their use before local excision. During the CARTS study, patients with cT1-3N0M0 rectal cancer admitted to referral centers for rectal cancer throughout the Netherlands were to be treated with neoadjuvant RT, followed by TEM in the case of a good response; the result was approximately two-thirds of patients had a good long-term oncological outcome and a high-rated quality of life (HRQL) [78]. A randomized trial by Lezoche et al. showed a similar local recurrence rate for TME (6%) as for TEM (8%) after neoadjuvant treatment in patients with ypT2 rectal cancer [79]. Neoadjuvant chemotherapy alone has been proposed in clinical trials. The Canadian Cancer Trials Group (CCTG) CO.28 NEO phase II trial was designed to determine the outcomes and organ preservation rate after the use of a preoperative folinic acid–fluorouracil-oxaliplatin 6 [mFOLFOX6] or capecitabine-oxaliplatin [CAPOX] course. These treatments resulted in a downstaging to ypT0/T1 cN0 in the majority of selected patients [80]; however, this treatment option potentially causes overtreatment since the clinical staging is not accurate in early rectal cancer. 

Since up to 30% of patients show a complete response to neoadjuvant CRT, rectal-sparing approaches (i.e., LE or watch-and-wait) were proposed to avoid surgery, for the management of selected patients with a complete clinical response (cCR) or near complete response (nCR) after neoadjuvant treatment [81]. A recent metanalysis comparing local excision and watch-and-wait approaches after neoadjuvant CRT showed no difference between watch-and-wait and LE when considering local disease, locoregional, and distant recurrence [82]. The neoadjuvant treatment strategy followed by LE is also associated with potential downsides. Local excision after neoadjuvant treatment impacts anorectal function and shows high rates of short-term morbidity, mostly due to pain, blood loss, and impaired wound healing [66]. 

### 6.2. Adjuvant Therapy

Following the local excision of high-risk pT1 and pT2 rectal cancer, an alternative treatment strategy involves the application of adjuvant chemoradiotherapy. This approach encompasses radiotherapy targeting the rectum and mesorectum, coupled with chemotherapy, aiming to diminish the likelihood of local recurrence. While adjuvant chemoradiotherapy itself carries some morbidity, serious complications generally remain within acceptable bounds [78]. Nevertheless, the conclusive results of randomized controlled trials, such as the TESAR trial, providing high-quality data and long-term outcomes for adjuvant chemoradiotherapy, are eagerly anticipated [15]. At present, the available evidence primarily relies on cohort studies featuring relatively small sample sizes. For instance a systematic review by Cutting et al. [83] revealed a 5.8% local recurrence rate for pT1 tumors and 13.8% for pT2 tumors. Additionally, a meta-analysis presented analogous findings but specified a local recurrence rate of 3.9% for high-risk pT1 tumors specifically, aligning with the outcomes of completion TME surgery [84]. One of the more extensive studies conducted by Jeong et al. [85] involved 83 patients and indicated a local recurrence rate of 3.6% in pT1 tumors. On the whole, adjuvant chemoradiotherapy emerges as an appealing option for rectum preservation while concurrently mitigating the risk of local recurrence when compared to a surveillance strategy. Nonetheless, substantiating its oncological safety necessitates the further accumulation of high-quality data and long-term oncological outcomes.

## 7. Future Perspectives

Early rectal cancer is a rising concern in terms of the increasing incidence in young adults and the burden of adverse events and side effects when performing TME. Local staging by an endoscopic optical evaluation and imaging techniques like high-resolution MRI, contrast-enhanced EUS, and PET-MRI association can now offer a precise local staging for early rectal cancer. Future advancement by the application of artificial intelligence algorithms to these diagnostic techniques can be expected to offer an even more accurate preoperative staging to select subsets of patients who could benefit from local excision techniques. Available local excision techniques are in continuous expansion with the introduction of new devices for both surgical and endoscopic techniques, making them more appealing and easier to use. Neoadjuvant CRT protocols and adjuvant CRT protocols have shown great efficacy in rectal cancer treatment and downstaging, and their combination with local excision techniques is opening a window for organ-sparing treatment even in cases where local excision techniques would not be indicated. For all these reasons, indications to perform local excision techniques are going to grow in the years to be, sparing a significant amount of morbidity linked to surgery in these patients.

## Data Availability

No new data were created or analyzed in this study. Data sharing is not applicable to this article.
